# Novel gene signature reveals prognostic model in acute lymphoblastic leukemia

**DOI:** 10.3389/fcell.2022.1036312

**Published:** 2022-11-03

**Authors:** Panpan Chen, Guanfei Gao, Yuanlin Xu, Peijun Jia, Yan Li, Yating Li, Jiaming Cao, Jiangfeng Du, Shijie Zhang, Jingxin Zhang

**Affiliations:** School of Life Sciences, Zhengzhou University, Zhengzhou, China

**Keywords:** acute lymphoblastic leukemia, prognostic value, risk model, bioinformatics analysis, database

## Abstract

Acute lymphoblastic leukemia (ALL) is a type of hematological malignancy and has a poor prognosis. In our study, we aimed to construct a prognostic model of ALL by identifying important genes closely related to ALL prognosis. We obtained transcriptome data (RNA-seq) of ALL samples from the GDC TARGET database and identified differentially expressed genes (DEGs) using the “DESeq” package of R software. We used univariate and multivariate cox regression analyses to screen out the prognostic genes of ALL. In our results, the risk score can be used as an independent prognostic factor to predict the prognosis of ALL patients [hazard ratio (HR) = 2.782, 95% CI = 1.903–4.068, *p* < 0.001]. Risk score in clinical parameters has high diagnostic sensitivity and specificity for predicting overall survival of ALL patients, and the area under curve (AUC) is 0.864 in the receiver operating characteristic (ROC) analysis results. Our study evaluated a potential prognostic signature with six genes and constructed a risk model significantly related to the prognosis of ALL patients. The results of this study can help clinicians to adjust the treatment plan and distinguish patients with good and poor prognosis for targeted treatment.

## Introduction

Acute lymphoblastic leukemia (ALL) is the most common childhood cancer and has a high incidence of mortality and low 5-year overall survival (OS) in patients who develop infection (Abdelmabood et al., 2020). Significant advances have been made in the treatment of childhood and adolescent ALL patients over the past few decades. More than 80% of patients diagnosed between the ages of 1 and 18 are expected to be long-term, event-free survivors. However, it is clear that further progress is required, as the survival rate of relapsed patients is not optimistic (Vrooman and Silverman, 2016).

Targeted tyrosine kinase inhibitors, tyrosine kinase inhibitors (TKI) targeting BCR-ABL1 tyrosine kinase, bispecific antibody blinatumomab and the antibody-drug conjugate inotuzumab ozogamicin, and chimeric antigen receptor (CAR)-T therapy are breakthrough approaches to treating ALL (Rafei et al., 2019; Gavralidis and Brunner, 2020). Great progress has been made in recent genome-wide study on leukemia cell DNA profiles. For instance, some researchers have found that methylation of p21 and p57 is associated with poor outcomes of ALL (Roman-Gomez et al., 2002; Shen et al., 2003). The co-inheritance of *IKZF1*, *ARID5B*, *CEBPE*, and *CDKN2A* was found to be a contributing factor to ALL in children (Yang et al., 2009; Sherborne et al., 2010). At present, treatment programs targeting different biological characteristics of ALL have achieved good curative effects, and the 5-year overall survival rate for children with ALL is approximately 90% (Hunger and Mullighan, 2015; Paul et al., 2016; Hefazi and Litzow, 2018; Horowitz et al., 2018; Cook and Litzow, 2020). Despite this, approximately 50% of adult patients and 20% of pediatric cases relapse after a period of treatment, showing that most adults with ALL still do not become long-term survivors (Fielding et al., 2007; Bhojwani and Pui, 2013; Locatelli et al., 2013). Therefore, it is crucial to obtain ALL prognosis-related genes to construct ALL prognostic gene signatures and build prognostic models to better predict treatment outcomes (Schultz et al., 2007; Mullighan et al., 2009; Kang et al., 2010; Cruz-Rodriguez et al., 2017). The present study was designed to build a prognostic model to predict the survival probability of ALL patients after receiving treatment, and provide a reference for clinicians to target ALL patients with different risk groups for treatment. We obtained RNA-seq data and compared the expression profiles between 89 bone marrow samples with ALL and 37 normal bone marrow samples. The dataset of ALL patients was downloaded from the GDC TARGET database in USCS Xena (https://doi.org/10.1038/s41587-020-0546-8) (Goldman and Craft, 2020). We identified 551 down-regulated genes and 461 up-regulated genes. We enriched the gene function using Kyoto Encyclopedia of Genes and Genomes (KEGG) pathway and Gene Ontology (GO) enrichment analyses. We employed univariate cox proportional hazard regression analysis to identify six prognostic related genes (*BAALC*, *HGF*, *CPXM1*, *CCL4*, *ZBTB10*, and *B3GNT2*) from the GDC TARGET ALL dataset, and constructed a risk score model by establishing the prognostic features of these genes. Next, we risk stratified patients according to their risk scores, performed survival analysis based on patients in the high-risk and low-risk groups, and performed ROC analysis for risk score, an independent prognostic factor. The results showed that patients in the high-risk group were strongly associated with poor prognosis, and the AUC value of the risk score was higher than other clinical parameters. To compare the differences in gene function between these two groups of patients, we performed Gene Set Enrichment Analysis (GSEA) analysis. Finally, to validate our constructed prognostic model, we downloaded RNA-seq data from 121 ALL patients from the International Cancer Genome Consortium (ICGC) database.

In this study, we evaluated the prediction results of risk score on the survival time of ALL patients by constructing a prognostic model. The results showed that risk score had a higher predictive value than other clinical parameters, such as age and gender. To the best of our knowledge, the AUC of risk score up to 0.864 in ROC analysis indicates that it has high specificity and sensitivity in predicting the survival time of ALL patients.

## Materials and methods

### The workflow for building the prognostic model

**FIGURE 1:**
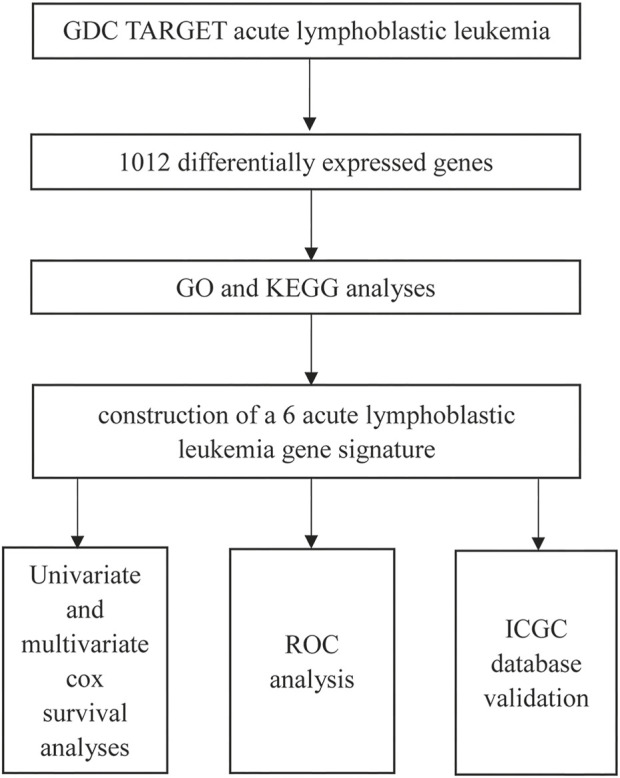
The workflow of the model construction process.

The workflow is shown in Figure 1.

### Sample of the study

The RNA-seq data and clinical information of 89 ALL patients and 37 bone marrow normal samples for control were downloaded from the UCSC Xena (cohort: GDC TARGET-ALL-P3) and The Cancer Genome Atlas (TCGA) database (https://portal.gdc.cancer.gov), respectively. GDC TARGET-ALL-P3 cohort contains 135 samples. We removed the sample ID ending with “B”, so there are 89 samples left. The purpose of downloading the independent TARGET ALL datasets (ALL-US Acute Lymphoblastic Leukemia—TARGET, US) from the ICGC database (https://dcc.icgc.org) was to validate our prognostic model.

### Functional annotation and GSEA

DEGs were identified using the packages of “tidyverse” and “DESeq” in R (version 4.1.2) with criteria of |log2fold-change (log2FC)| > 1 and adjusted *p*-value < 0.05. GO and KEGG analyses were performed using the packages of “tidyverse”, “BiocManager”, “org.Hs.eg.db”, and “clusterProfiler” in R (Wu et al., 2021). KEGG and GO including three categories of biological process (BP), molecular function (MF), and cellular component (CC) enables efficient clustering of functional genes (Chen et al., 2017; Kanehisa et al., 2017). GSEA (Subramanian et al., 2005) was performed by using the packages of “tidyverse”, “ReactomePA”, “data.table”, “org.Hs.eg.db”, “clusterProfiler”, “biomaRt”, and “enrichplot” in R.

### Construction of the six prognostic genes signature

Univariate cox analysis was performed by using the “tidyverse”, “survival” and “forestplot” packages in R. Then LASSO logistic regression analysis (R package, glmnet, v4.1-4) was used to further screen genes associated with the prognosis of ALL. Six prognostic genes were identified based on *p* < 0.05. Kaplan-Meier survival analysis and log-rank test were performed by the packages of “tidyverse”, “survival” and “survminer” in R to verify whether these six genes were associated with ALL prognosis (George et al., 2014). Next, a genetic prognostic signature was constructed based on multivariate cox regression analysis by using the “tidyverse” and “survival” packages in R (Cioci et al., 2021). The risk score model was performed using the packages of “tidyverse”, “survival”, and “ggrisk” in R.

### ROC analysis

ROC analysis (Obuchowski, 2005; Hoo et al., 2017) and the calculation of the area under the curve (AUC) were analyzed by the “tidyverse”, “ROCR”, and “rms” packages of R software. The risk score and other clinical factors correlated with survival were used in Multi-index ROC analysis. A *p*-value < 0.05 was considered as statistical significance.

All statistical analyses in this study were performed by R (version 4.1.2). *p* < 0.05 was considered of statistical significance.

## Results

### Clinical information for ALL patients

Of the 89 patient samples we obtained from the GDC TARGET database, 69 patients had clinical information. The clinical information of the patients are listed in Table 1, including white blood cell (WBC) count at diagnosis (×10^9^/L), vital status, and initial therapy. Among them, 28 (40.6%) were female and 41 (59.4%) patients were male. The age at initial diagnosis ranged from 0.1 to 17.9 years. In initial therapy, 26 (37.7%) patients were ALL, 27 (39.1%) patients were acute myeloid leukemia (AML), and 7 (10.1%) patients were hybrid.

**TABLE 1:** Characteristic of ALL patients.

Characteristic	Number of cases	Percentages (%)
Patients	69	100
Age at diagnosis (year)		
Median	8.1	—
Range	0.1–17.9	—
Sex		
Female	28	40.6
Male	41	59.4
WBC count at diagnosis (×10^9^/L)		
≥50	30	43.5
<50	33	47.8
NA	6	8.7
Vital status		
Alive	42	60.9
Dead	27	39.1
Initial therapy		
ALL	26	37.7
AML	27	39.1
Hybrid	7	10.1
Interfant06	1	1.4
Unknown	8	11.6

### Identification of differentially expressed genes

**FIGURE 2:**
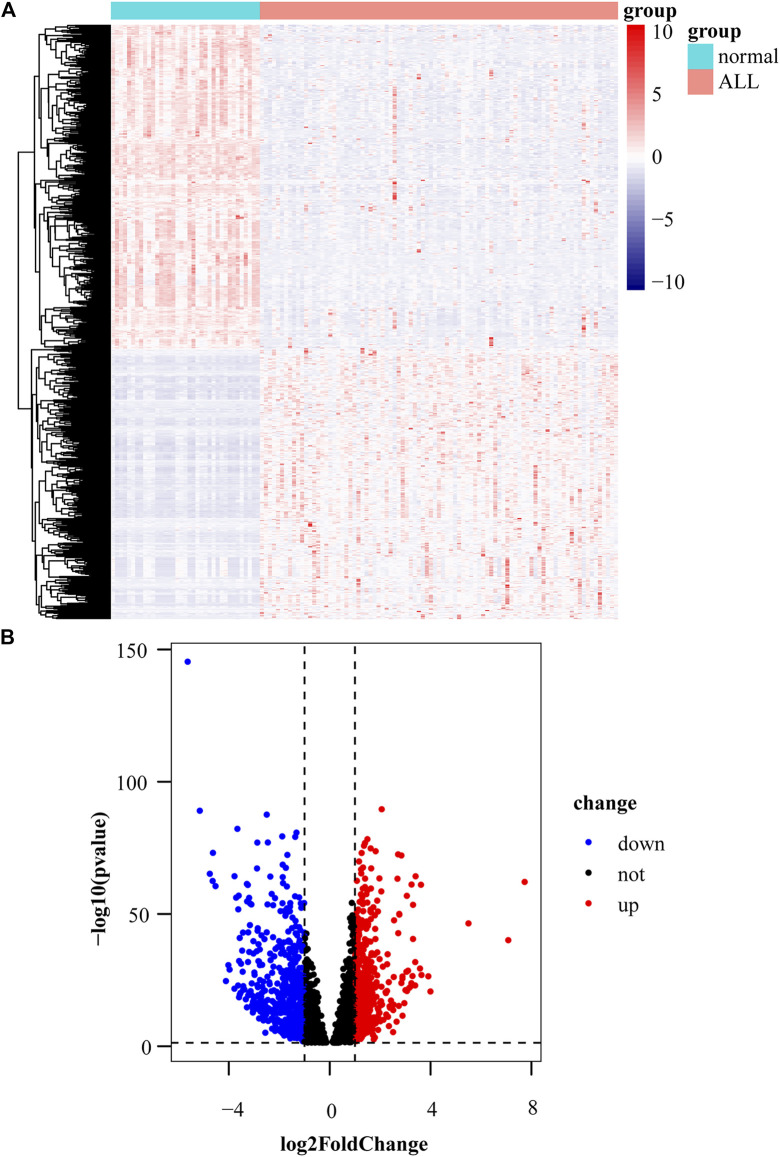
Identification of differentially expressed genes. **(A)** Heat map of differentially expressed gene expression levels in the GDC TARGET-ALL dataset. **(B)** Volcano plot for differentially expressed gene expression levels in GDC TARGET-ALL dataset.

We obtained 551 down-regulated genes and 461 up-regulated genes among the 1012 DEGs we obtained. These results are presented as a heat map by the “tidyverse” and “pheatmap” packages of R software (Figure 2A) and a volcano plot using the packages of “tidyverse”, “ggpubr” and “ggthemes” in R (Figure 2B). We screened the top 10 up-regulated and down-regulated genes according to |log2FC|, as shown in Tables 2, 3.

**TABLE 2:** The top 10 up-regulated genes.

Gene	BaseMean	Log2FoldChange	IfcSE	Stat	*p*-value	*P*-adjust
*MYO18B*	4403.147	7.732	0.462	16.733	7.586E-63	7.368E-61
*S100A16*	508.046	7.083	0.529	13.378	8.159E-41	1.829E-39
*CCNA1*	850.673	5.503	0.382	14.425	3.625E-47	1.184E-45
*SUCNR1*	1868.296	3.996	0.420	9.504	2.019E-21	1.216E-20
*BAALC*	1743.437	3.913	0.362	10.794	3.668E-27	3.518E-26
*PROM1*	3187.455	3.641	0.334	10.898	1.172E-27	1.175E-26
*FLT3*	9389.460	3.620	0.218	16.584	9.095E-62	8.012E-60
*RFX8*	754.736	3.582	0.314	11.400	4.169E-30	4.919E-29
*SOX4*	10003.947	3.406	0.200	17.022	5.676E-65	6.936E-63
*SPRY1*	1579.886	3.393	0.286	11.877	1.557E-32	2.136E-31

**TABLE 3:** The top 10 down-regulated genes.

Gene	BaseMean	Log2FoldChange	IfcSE	Stat	*p*-value	*P*-adjust
*IL2RB*	2067.055	−5.632	0.219	−25.739	4.258E-146	1.612E-142
*FCRL6*	382.885	−5.151	0.256	−20.089	9.288E-90	1.173E-86
*GNLY*	3373.315	−4.754	0.277	−17.148	6.483E-66	8.468E-64
*GZMH*	526.780	−4.641	0.277	−16.782	3.290E-63	3.280E-61
*FGFBP2*	533.131	−4.630	0.255	−18.176	7.990E-74	1.892E-71
*GZMB*	773.903	−4.527	0.274	−16.512	2.992E-61	2.519E-59
*HBM*	1754.903	−4.114	0.395	−10.406	2.339E-25	1.926E-24
*CA1*	7487.588	−4.019	0.345	−11.654	2.183E-31	2.747E-30
*KCNH2*	1079.042	−3.970	0.351	−11.321	1.037E-29	1.205E-28
*AHSP*	2477.869	−3.779	0.388	−9.750	1.837E-22	1.196E-21

### GO enrichment and KEGG pathway analysis

**FIGURE 3:**
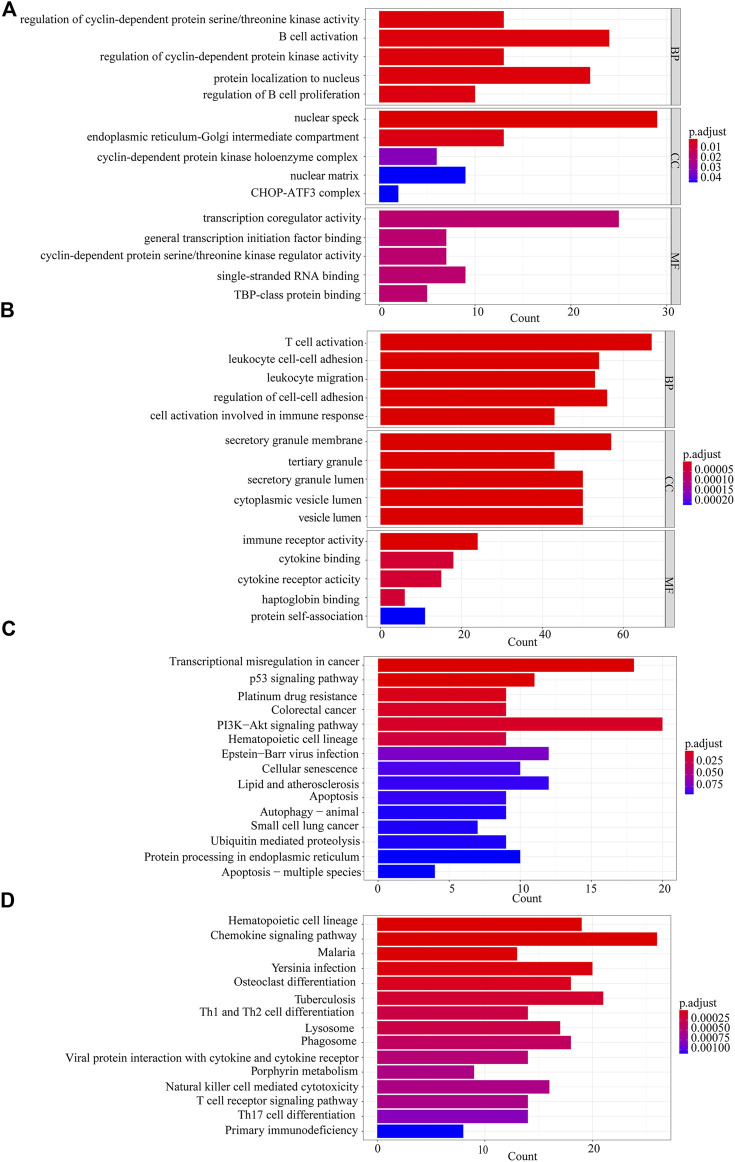
Gene Ontology (GO) enrichment and Kyoto Encyclopedia of Genes and Genomes (KEGG) pathway analyses. **(A)** The GO analysis of 461 up-regulated genes. **(B)** The GO analysis of 551 down-regulated genes. **(C)** KEGG analysis of 461 up-regulated genes. **(D)** KEGG analysis of 551 down-regulated genes.

We performed GO and KEGG analyses on the 1012 DEGs and select enriched results with a significance level at adjusted *p*-value < 0.05. In GO enrichment analysis of the up-regulated genes, the mainly enriched terms were B cell activation, protein localization to nucleus, intrinsic apoptotic signaling pathway, and nuclear speck (Figure 3A). The down-regulated genes were enriched in T cell activation, regulation of cell-cell adhesion, secretory granule membrane, and immune receptor activity (Figure 3B). The top pathways enriched for up-regulated genes and down-regulated genes in KEGG analysis were PI3K-Akt signaling pathway and chemokine signaling pathway, respectively (Figures 3C,D).

### The affection of the six prognostic genes on survival in ALL patients

**FIGURE 4:**
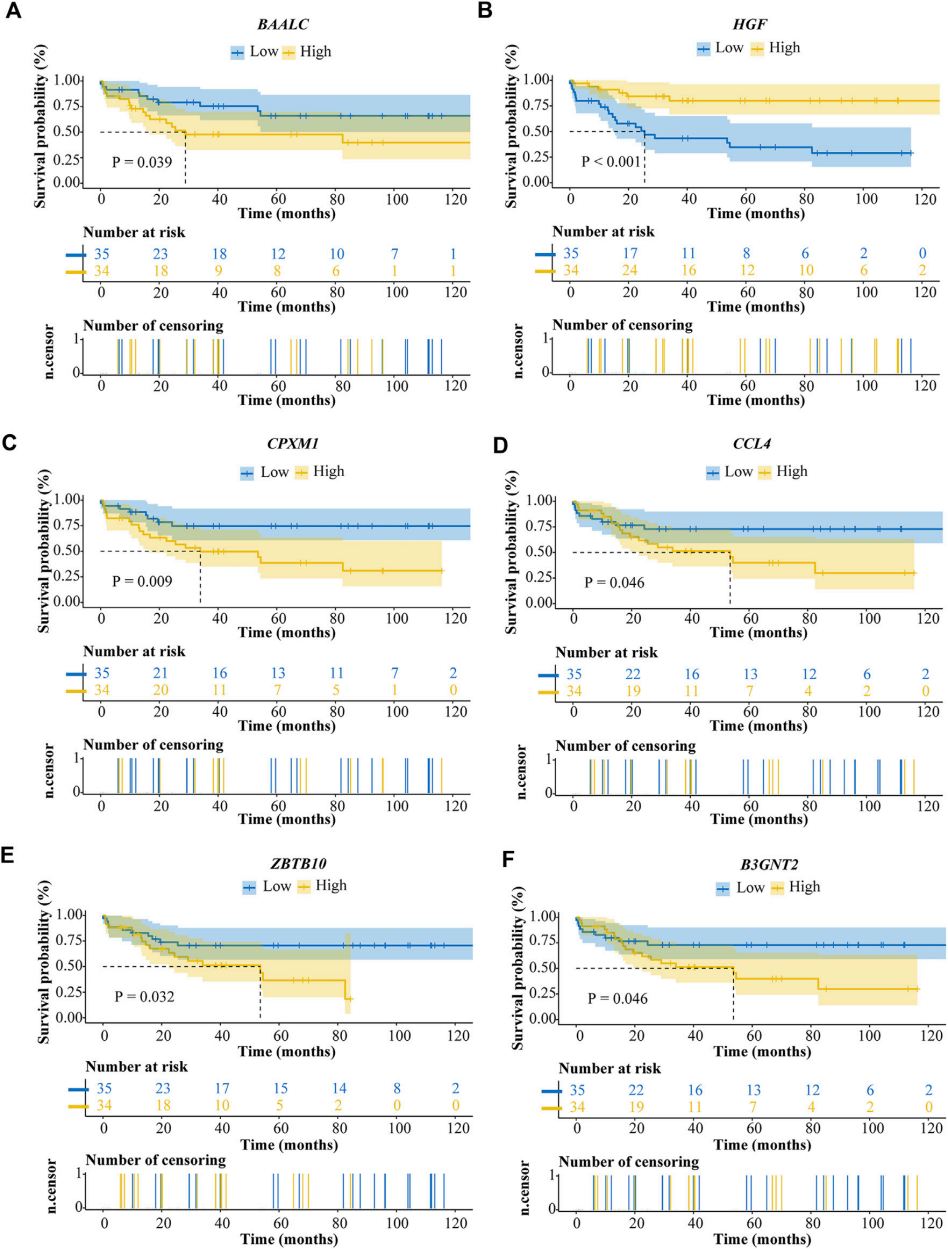
Survival analysis of ALL prognostic genes. **(A)** *BAALC*. **(B)** *HGF*. **(C)** *CPXM1*. **(D)** *CCL4*. **(E)** *ZBTB10*. **(F)** *B3GNT2*.

**FIGURE 5:**
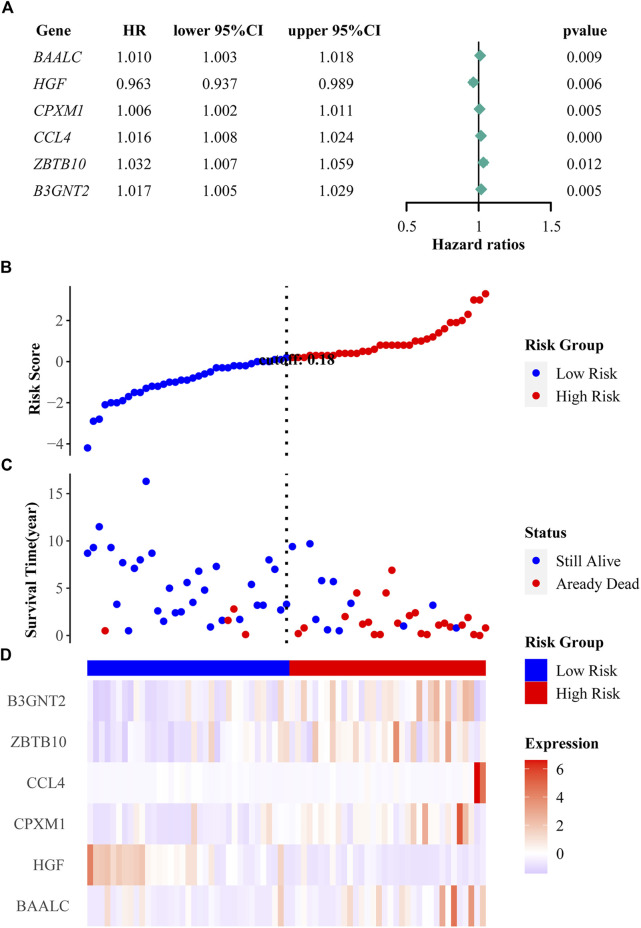
Regression analysis and characteristics of prognostic gene signatures. **(A)** Forest map of six prognostic genes by univariate cox regression. **(B)** Distributions of risk scores in all samples. **(C)** Distribution of follow-up times in the training samples. **(D)** Heat map of gene expression in the prognostic signature of ALL.

To identify which genes are associated with ALL, we performed univariate cox and LASSO logistic regression analyses and then obtained a most stable gene set with six genes (Figure 5A). The genes that have been identified are *BAALC*, *HGF*, *CPXM1*, *CCL4*, *ZBTB10*, and *B3GNT2*. To validate the potential values of the six genes predicting prognosis, we performed survival analysis by the “tidyverse”, “survival” and “survminer” packages of R software. The results showed that the six prognostic genes significantly impact the overall survival by using a log-rank test (*p* < 0.05). Highly expressed *BAALC*, *CPXM1*, *CCL4*, *ZBTB10*, and *B3GNT2* are associated with poor prognosis. The low expression of *HGF* indicates that it is a protective gene in ALL patients (Figures 4A–F).

### Establishment of the prognostic risk model

We constructed the risk score model through the calculation formula (Qu et al., 2020): risk score = (0.01233195 × expression level of *BAALC*) + (−0.03008332 × expression level of *HGF*) + (0.00458174 × expression level of *CPXM1*) + (0.01487359 × expression level of *CCL4*) + (0.00933869 × expression level of *ZBTB10*) + (0.01274584 × expression level of *B3GNT2*). The coefficients of the six prognostic genes are shown in Table 4. The number of deaths in the high-risk group was significantly higher than that in the low-risk group (Figures 5B,C). The expression of these six prognostic genes in these two distinct populations is shown in Figure 5D.

**TABLE 4:** Genes included in prognostic ALL genes signature.

Gene	Coef	HR	Z	*p*-value	Lower	Upper
*BAALC*	0.012332	1.012408	2.634728	0.008420	1.003163	1.021739
*HGF*	−0.030083	0.970365	−2.122150	0.033825	0.943775	0.997704
*CPXM1*	0.004582	1.004592	1.529872	0.126048	0.998713	1.010506
*CCL4*	0.014874	1.014985	3.278652	0.001043	1.006000	1.024050
*ZBTB10*	0.009339	1.009382	0.515572	0.606154	0.974177	1.045861
*B3GNT2*	0.012746	1.012827	1.797893	0.072194	0.998852	1.026999

### The genetic risk score model is an independent prognostic factor

**FIGURE 6:**
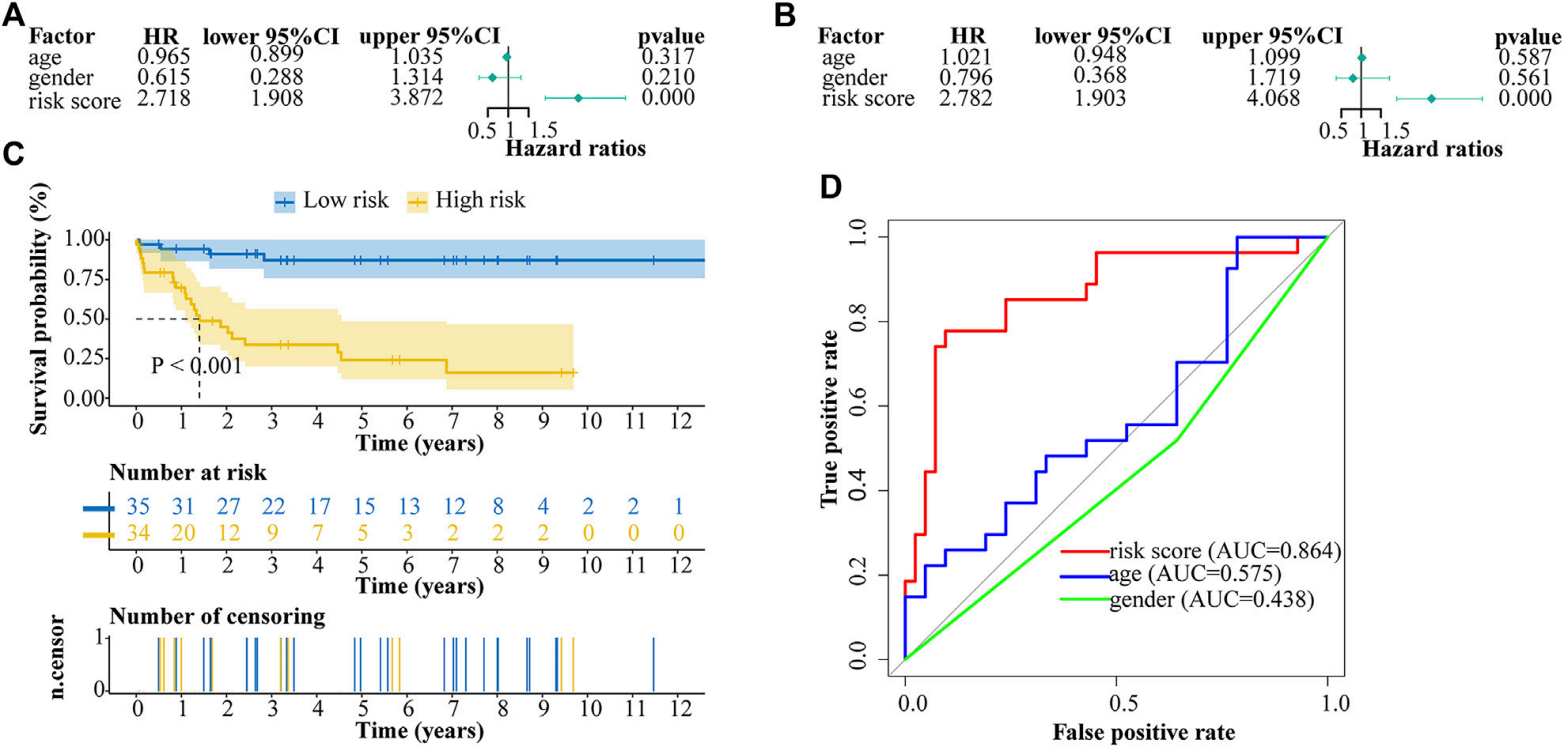
Cox proportional hazard regression analysis of ALL risk factors. **(A)** Univariate cox regression analysis of risk score and other indicators. **(B)** Multivariate cox regression analysis of risk score and other indicators. **(C)** Kaplan-Meier analysis of ALL patients grouped according to median risk. **(D)** Multi-index receiver operating characteristic (ROC) curve of risk score and other indicators.

We performed univariate and multivariate cox analyses to evaluate the prognostic value of risk score and other clinical parameters. Univariate cox regression analysis showed that the risk score was significantly correlated with overall survival (OS) (HR = 2.718, 95% CI = 1.908–3.872, *p* < 0.001) (Figure 6A). Multivariate cox regression analysis proved the risk score could be an independent factor predicting the prognosis of ALL patients (HR = 2.782, 95% CI = 1.903–4.068, *p* < 0.001) (Figure 6B). OS rate was significantly lower in high-risk patients compared with low-risk patients (Figure 6C). Multi-ROC result revealed that the AUC of risk score was 0.864 which was the highest one than the AUCs of age (0.575) and gender (0.438) (Figure 6D).

### GSEA of the prognostic signature in the low-risk and high-risk groups

**FIGURE 7:**
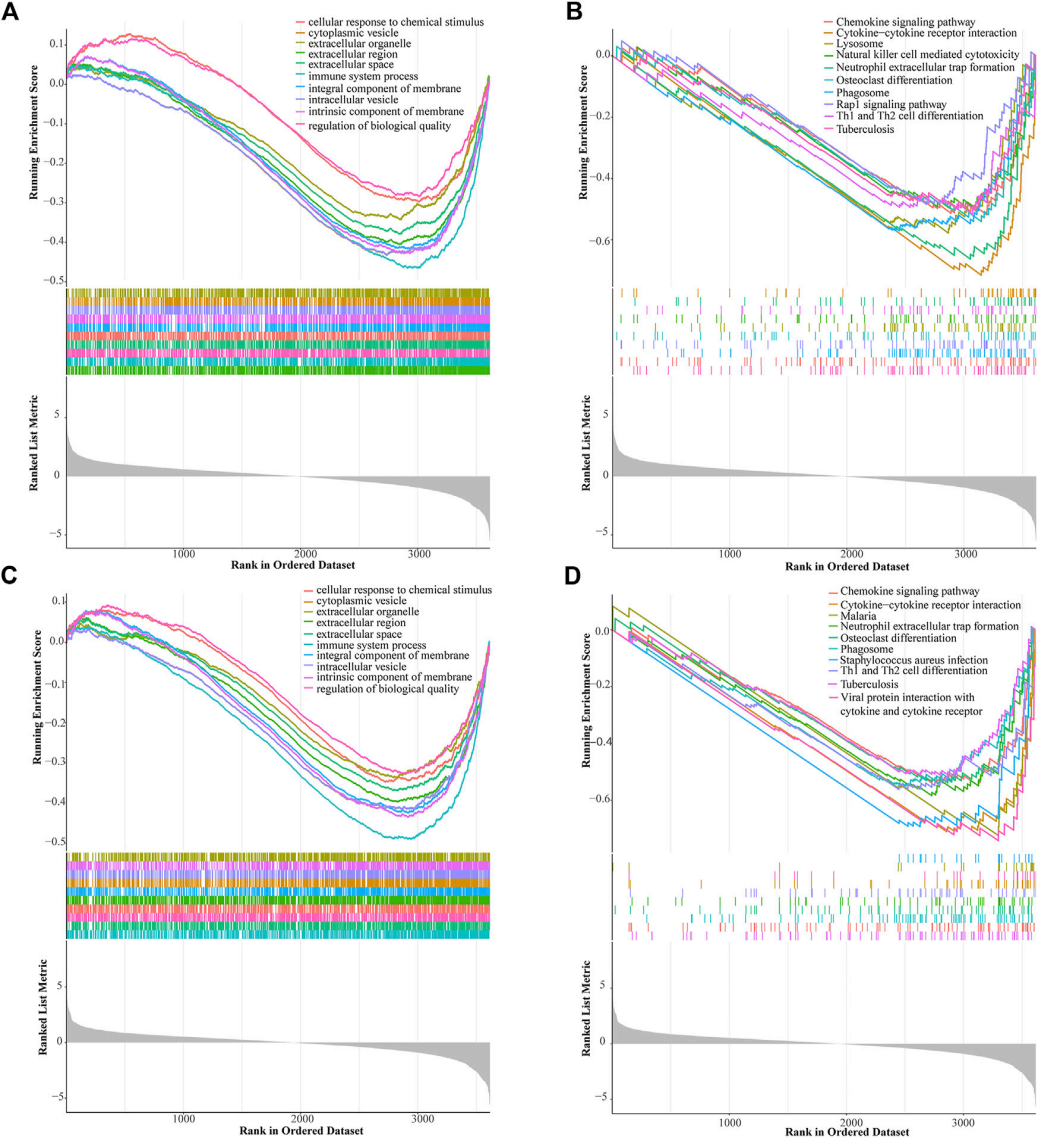
Gene set enrichment analysis (GSEA) of the prognostic signature. GSEA shows the GO **(A)** and KEGG pathways **(B)** enriched in the high-risk group of the ALL gene signatures. GSEA reveals the GO **(C)** and KEGG pathways **(D)** enriched in the low-risk group of the ALL gene signatures.

We used the GSEA to identify potential signaling pathways in the low-risk and high-risk groups (Figures 7A–D). Among the top 10 pathways of KEGG in the low-risk and high-risk groups, there were three significantly different pathways, namely lysosome, natural killer cell mediated cytotoxicity, and Rap1 signaling pathway.

### External verification of the prognostic gene signature

**FIGURE 8:**
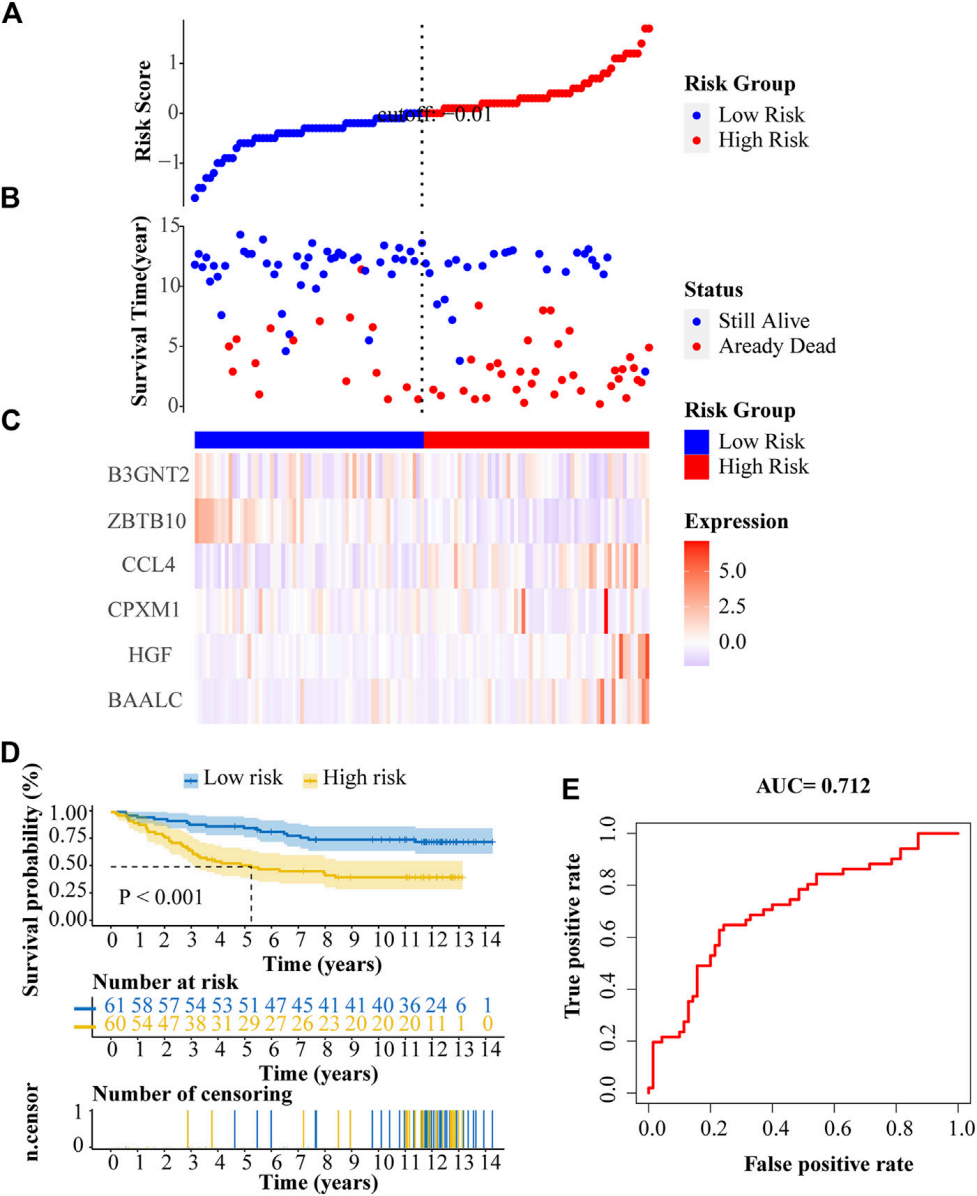
External verification of the prognostic ALL gene signature. **(A)** Risk scores distribution. **(B)** Survival status of patients. **(C)** Heat map of gene expression pattern. **(D)** Kaplan-Meier plot for overall survival (OS) of patients in different risk groups. **(E)** ROC curve of risk score.

We validated our prognostic model with an independent ALL dataset downloaded from the ICGC database. The distribution of risk score, survival status, and gene expression patterns of patients are shown in Figures 8A–C. The high-risk patients had a significantly poorer OS than patients in the low-risk group (Figure 8D). In multi-ROC analysis, the AUC of risk score was 0.712, indicating high diagnostic sensitivity and specificity for patients OS (Figure 8E).

## Discussion

In this study, we obtained 89 bone marrow samples with ALL and 37 normal bone marrow samples from the GDC TARGET database. We analyzed the RNA expression profiles of these samples and identified 1012 DEGs. By GO and KEGG analyses, we found that up-regulated genes were mainly enriched in B cell activation and PI3K-Akt signaling pathway. Interestingly, previous studies demonstrated that B-cell malignancies result from abnormal dysregulation of B-cell–activating factor (BAFF) (Li et al., 2021) and the PI3K-Akt signaling pathway in B-lineage ALL is activated through the interaction of PI3K-p85 and CD9 (Shi et al., 2021). The AUC of the risk score was greater than 0.7, indicating that the risk score has a good prognosis predictive value for more than 70% of ALL patients (Huang et al., 2020). GSEA analysis in high-risk group showed neutrophil extracellular trap formation and Rap1 signaling pathway were highly associated with ALL. Previous researches have shown that median neutrophil elastase activity and neutrophil extracellular traps (NETs) formation are higher in ALL versus acute myeloid leukemia (Berger-Achituv and Elhasid, 2018) and Notch-dependent T-ALL results from dysregulated constitutive Rap1 activation (Wang et al., 2008).

In this study, we identified six genes (*BAALC*, *HGF*, *CPXM1*, *CCL4*, *ZBTB10*, and *B3GNT2*) associated with ALL prognosis. In Multi-index ROC analysis of the training set, the AUC of risk score was 0.864, which was the highest one than that of other clinical parameters. This indicates that the prognostic model we constructed has high predictive value. However, our study also has limitations. First, we were unable to collect sufficient ALL cases. Second, the mechanism of ALL prognostic genes requires further study.

Among the six ALL prognostic genes, *BAALC* has been reported to be associated with ALL. Diseases associated with *BAALC* include acute leukemia and leukemia. Previous study demonstrated that high *BAALC* expression contributes to poor prognosis in childhood ALL (Azizi et al., 2015). There were significant differences in disease outcomes between *BAALC*-positive and *BAALC*-negative groups, and the positive group had higher recurrence and mortality rates (Hagag et al., 2018). A previous study showed that patients with an immature, chemoresistant leukemic phenotype have higher *BAALC* expression and poorer OS (Kühnl et al., 2010). The HGF/c-MET signaling pathway is involved in the occurrence and progression of hematological tumors including T-cell acute lymphoblastic leukemias (Accordi et al., 2007). *HGF* levels in patients with acute leukemia are higher than those in healthy individuals, and higher *HGF* expression is associated with lower survival rates (Kim et al., 2005). A previous study showed that *CPXM1* is closely associated with leukemia stem cells and can be used as a prognostic gene in patients with acute myeloid leukemia (AML) and myelodysplastic syndrome (MDS) (Wang et al., 2020). The polymorphism of *CCL4* gene affects gene expression and protein function, and has been confirmed to predict the risk and prognosis of a variety of diseases (Kuo et al., 2018). *ZBTB10* can be down-regulated by MicroRNA-27a (miR-27a) to promote tumor growth and metastasis (Tang et al., 2012). Upregulation of *B3GNT2*, which encodes a poly-N-acetyllactosamine synthase, allows human melanoma cells to evade T cell killing in different cancer cell types, and can reduce T cell activation and disrupt the interaction between tumor and T cells (Joung et al., 2022). Although *CPXM1*, *CCL4*, *ZBTB10* and *B3GNT2* have not been reported to be related to the prognosis of ALL, the four genes we screened are all closely related to cancer, and we have reason to believe that they may also be potential genes for predicting the prognosis of ALL.

Based on these six genes, we constructed the prognostic model and divided ALL patients into high-risk and low-risk groups. Different risk groups show different characteristics, which helps clinicians to make targeted treatment. Risk-stratified therapy has steadily improved survival while reducing treatment-related toxicity and has made steady progress in improving survival in children with ALL (Mattano and Devidas, 2021). In addition, the cure rate of ALL in high-income countries is high, but it does not appear in low and middle-income countries (LMIC). 80% of the world childhood ALL burden occurs in LMIC (Oh et al., 2021). Risk stratification treatment can significantly reduce the treatment burden for patients with ALL in LMIC.

In summary, we constructed a prognostic model of ALL by identifying six genes closely related to ALL prognosis. Our findings will provide a reference for clinicians to choose personalized treatment options and provide potential drug targets for the treatment of ALL.

## Data Availability

Publiclyavailable datasets were analyzed in this study. This data can be found here: The datasets (cohort: GDC TARGET-ALL-P3): UCSC Xena at https://xenabrowser.net/datapages/; bone marrow normal samples: The Cancer Genome Atlas (TCGA) database at https://portal.gdc.cancer.gov; independent TARGET ALL datasets (ALL-US Acute Lymphoblastic Leukemia—TARGET, US): the International Cancer Genome Consortium (ICGC) database at https://dcc.icgc.org.
